# Workplace Accommodations and Employment Outcomes Among Employees With Autism: A Systematic Review

**DOI:** 10.7759/cureus.99353

**Published:** 2025-12-16

**Authors:** Cécile Heinze

**Affiliations:** 1 Behavioral Health, AutiSoul, Miami, USA

**Keywords:** autism, employment rate, vocational rehabilitation, workplace, workplace accommodations

## Abstract

Individuals with autism often face challenges in achieving and maintaining meaningful employment, but workplace accommodations can bridge these gaps by enabling inclusion, satisfaction, and productivity. The goal of this review was to systematically evaluate and synthesize the existing evidence base on workplace accommodations for adults with autism. After conducting a systematic search of numerous databases, ten empirical studies published between 2010 and 2025, including randomized controlled trials, cohort studies, surveys, and qualitative studies, were included. The quality of studies was assessed using the Critical Appraisal Skills Programme (CASP), the Joanna Briggs Institute (JBI), and Risk-of-Bias (RoB) instruments, with the majority of studies being assessed as moderate-to-high quality but having small sample sizes and relying on self-report. Accommodation reports fall into four groups: assistive technology; organizational, sensory, and environmental adaptations; supervisory and relational support; and skills and psychosocial support. In general, the evidence indicated that accommodations were linked to better job acquisition, stability, satisfaction, and productivity, albeit with variability in effectiveness based on relational quality, disclosure practice, and organizational culture. Consistent results highlighted the value of individually tailored support and respectful supervisory relationships, while variations indicated lingering barriers to stigma, ignorance, and uneven policy implementation. However, the heterogeneity of methods and the exclusion of broader autism groups limit the generalizability of findings in these studies. Longitudinal and intervention studies, participatory approaches to design, and representation of underrepresented groups are essential priorities for future research to further develop evidence-based practice and guide employer training and policy.

## Introduction and background

The American Psychiatric Association [1] defines autism spectrum disorder (ASD) as a developmental illness that is characterized by repetitive behaviors or interests and a lack of social interaction and communication. The American Psychiatric Association [1] claims that it is a spectrum condition that can be perceived as weaker or more severe, and in the latter case, individuals may need support to be able to act independently. Throughout their lives, individuals with ASD regularly encounter significant challenges at work [2,3]. Such barriers are often instigated by resource and support shortages and opinions. The autistic community is highly diverse, as it can have different distinctive qualities (e.g., auditory processing disorder, interoception, echolalia, alexithymia) and different support needs, so it cannot be served by a single strategy that suits everyone [4]. As a result, prominent organizations' neurodiversity projects mostly focus on autism [5,6]. Despite these efforts, the employment rate for those with autism is lower than that of individuals with other disabilities, including learning difficulties, speech, language, and vision impairments [7]. A growing neurodiversity-based advocacy movement among autistic people contends that autism is only one form of human neurological expression and should not be the focus of attempts at normalization or treatment. To better integrate autistic people into our societies, neurodiverse adherents advocate for broader societal acceptance and comprehension of autistic differences. The principles of neurodiversity are essential for helping autistic people develop positive disability identities, as autism is viewed as being crucial to identity development in this approach. Person-first language employed in other disability groups is reflected in the preference of autistic self-advocates for the phrase "autistic person" over "person with autism" [8-10].

Post-secondary results for autistic people remain depressing when compared to adults without disabilities and adults in other disability groups, despite greater activism and public awareness of autism [11]. Overall, the employment rates of individuals with impairments are significantly lower than those of people without disabilities [12]. Despite initiatives to lessen workplace discrimination outlined in the Americans With Disabilities Act (ADA), these tendencies persist. In what has been referred to as the "spirit" of the legislation, scholars have observed that these attempts have not been successful in changing attitudes that hinder employment [13,14]. Although there are differences in the reported employment rates among autistic individuals, the employment rate for this group has often been around 50% [15,11,16]. A vital human right that plays a significant role in people's lives is employment [17]. Rehabilitation professionals often seek to enhance the employment and retention of individuals with disabilities [18]. According to Dutta et al. [19], engaging in competitive and fulfilling work is essential for the physical and mental health of both those with and without impairments. Employment can enhance social networks, mental health, and social inclusion [20,21]. Conversely, unemployment is associated with a worse quality of life and a higher frequency of anxiety and sadness [19]. Around the world, employment rates for autistic people are low (e.g., Australia, 27.3%; Israel, 28%; the United Kingdom, 29%; the United States and Canada, 14% [22-26]). Even when employed, autistic people often experience underemployment and lower earnings [27]. Only about 25% of autistic adults achieve what can be considered satisfactory occupational and social outcomes [28-30], although there is strong evidence of heterogeneity in population study findings [31].

Education level has consistently correlated with enhanced outcomes; however, individual factors such as gender, ethnicity, and education have exhibited divergent effects [32-34]. While co-occurring disorders and behaviors of concern frequently predict worse results, autism-specific variables, such as adaptive functioning and later age of diagnosis, have been linked to better work chances [35,23,36]. Although there is conflicting evidence on their long-term effects, family factors such as parental education, employment attitudes, and socioeconomic status may further modify opportunities [33,37,38]. Because they promote social support and workplace changes, vocational aids, including counselling, targeted job training, and disclosing a diagnosis to employers, have consistently shown favorable results [39,34,40]. Past research has identified several barriers that influence the working lives of autistic people, with six specific factors being most salient: difficulties in communication and social interaction, stress management deficits, executive function difficulties, motor or movement differences, and sensory sensitivity [41,42]. Different ways of communicating can lead to misunderstandings during hiring and at work, which are often wrongly blamed on rudeness or lack of interest. Such issues can affect getting and keeping a job [43]. Stigma from society and negative attitudes also limit access to necessary accommodations, resulting in internalized stigma and decreased job satisfaction [44,45]. Stress management problems, especially masking and excessive social anxiety, are associated with vocational disability and mental burnout [46,47].

Executive function deficits, such as working memory and impaired flexibility, can hinder task organization and decision-making, despite frequent autistic strengths in structured problem-solving [48]. Coordination difficulties of the motor type, along with sensory sensitivities and repetitive behaviors, add complexity to workplace transitions, especially in overstimulating settings [49,50]. In combination, these barriers demonstrate the need for sensory-friendly spaces and inclusive actions to maximize employment outcomes for autistic individuals [51]. Accommodation in the workplace is essential in assisting autistic workers since it may alleviate obstacles, improve job satisfaction, and increase retention if implemented effectively [52,53]. Individualized adaptations, e.g., sensory adaptations, quiet rooms, and adaptable timetables, support autistic employees in their productivity and welfare while acknowledging their specific requirements and strengths [43,54]. Challenges, however, occur when autistic individuals fail to recognize or ask for accommodations, especially in cases where they have been masking and later disclose, as this is bound to be stigmatized or risk discrimination [55,56]. Employers might inaccurately believe accommodations are expensive or unfair, though data indicate most adaptations are cheap and equally beneficial [57,58]. In addition, poor follow-through on requests for accommodations has the potential to prolong stress and turnover among autistic employees [59]. To create a culture of inclusion, organizations should encourage disability awareness training, open policies, and collaborative strategies that enable autistic staff while correcting employer misconceptions [60,61].

In spite of growing research focus on employment and autism, most of the evidence up to now has focused on pre-employment preparation, vocational training, or individual-level predictors rather than conducting a limited systematic exploration of the effect of workplace accommodation on employment outcomes for autistic adults in competitive, paid positions. The heterogeneity of this literature, coupled with variability in methodologies and outcome measures, has precluded strong conclusions for policy and practice. In order to address this, the current systematic review combines existing evidence on workplace accommodations and employment outcomes among autistic workers. The aim is to deliver a well-researched, evidence-based evaluation that can inform employers, service providers, policymakers, and researchers in developing inclusive workplace interventions that facilitate sustainable and meaningful work for autistic adults.

## Review

Methodology

Protocol and Reporting Standards

This systematic review followed the Preferred Reporting Items for Systematic Reviews and Meta-Analyses (PRISMA) 2020 statement [62]. The protocol was created prospectively, with preselected eligibility criteria, search terms, and appraisal strategies. While not formally registered with the International Prospective Register of Systematic Reviews (PROSPERO), procedures conformed to international best practices to maximize transparency and reproducibility. Decisions made throughout the process, including modifications to search terms and inclusion criteria, were recorded to ascertain methodological consistency. A narrative synthesis method was employed to summarize and explain the results because the research included different methods and ideas. The extracted results were categorized into principal outcome groups to facilitate theme integration. A meta-analysis was impractical because of significant variability in trial designs, outcome measurements, and analytical methodologies; hence, no aggregated effect estimates or statistical models were generated.

Search Strategy

A systematic search of the literature was conducted to locate empirical studies on workplace accommodations and employment outcomes of autistic workers. The following electronic databases were searched between January 2010 and February 2025: PubMed, Cochrane Library, Cumulative Index to Nursing and Allied Health Literature (CINAHL), Medical Literature Analysis and Retrieval System Online (MEDLINE) (ProQuest), and PsycINFO. Search strategies intersected controlled vocabulary terms (e.g., MeSH headings) with free-text keywords from three domains: population (autistic adults, autism spectrum disorders, and Asperger's); intervention (workplace accommodations, reasonable adjustments, assistive technology, job coaching, and sensory or organizational supports); and outcomes (employment, job satisfaction, retention, performance, and workplace inclusion). Search strings were specifically modified per database to be as sensitive as possible (Table 1). No limits were imposed at the initial level except for date and language filters. To guarantee thoroughness, lists of included studies and previous reviews were searched manually for extra records. All the retrieved citations were electronically imported into EndNote X9 (Clarivate, London, UK), and duplicates were deleted. Rayyan software (Rayyan Systems Inc., Cambridge, MA) was subsequently employed to aid blinded screening and decision tracking [63]. All included studies were thoroughly assessed using the Risk-of-Bias (RoB) 2.0 tool for randomized trials [64]; the Critical Appraisal Skills Programme (CASP) Cohort and Qualitative Checklists for longitudinal and interview studies [65,66]; and the Joanna Briggs Institute (JBI) critical appraisal checklists for cross-sectional surveys and descriptive studies [67]. A narrative synthesis was conducted in accordance with established guidance for synthesizing diverse types of evidence [68].

**Table 1:** Search terms CINAHL: Cumulative Index to Nursing and Allied Health Literature; MEDLINE: Medical Literature Analysis and Retrieval System Online

Databases	Strings
PubMed	(("Autism Spectrum Disorder"[Mesh] OR autis*[tiab] OR asperger*[tiab] OR ASD[tiab] OR "autistic employees"[tiab] OR "autistic workers"[tiab]) AND ("Reasonable Accommodation"[tiab] OR "reasonable accommodation"[tiab] OR "workplace accommodations"[tiab] OR "job accommodations"[tiab] OR "reasonable adjustments"[tiab] OR "workplace support*"[tiab] OR "occupational adjustment*"[tiab] OR "inclusive employment"[tiab] OR "workplace inclusion"[tiab] OR "disability support"[tiab]) AND ("Employment"[Mesh] OR employ*[tiab] OR "employment outcome*"[tiab] OR "job satisfaction"[tiab] OR "employee well-being"[tiab] OR retention[tiab] OR performance[tiab] OR "career progression"[tiab]))
Cochrane Library	((((autism) OR (autistic employees)) OR (ASD)) AND (((workplace accommodations) OR (job accommodations)) OR (reasonable adjustments))) AND ((((employment outcomes) OR (job satisfaction)) OR (retention)) OR (performance))
CINAHL	(MM "Autism Spectrum Disorder" OR MM "Asperger Syndrome") OR autism OR (autism or asd or autism spectrum disorder or asperger's or asperger's syndrome or autistic disorder or aspergers) AND XB (workplace accommodation) OR XB (workplace accommodation or job accommodation) OR MJ (reasonable adjustments or reasonable accommodations) OR MJ (occupational adjustment) OR MJ (workplace support or occupational support) OR TI (inclusive employment) AND MW (employment outcomes) OR MJ (employment or jobs or work or career or workplace or work environment or work spaces or office or job or work culture or corporate culture) OR SU (job satisfaction or work satisfaction or employee satisfaction or turnover or retention or attrition) OR TX (employee well being in the workplace) OR MW (performance or productivity or efficiency or success or outcomes) OR MJ (career progression or promotion or career advancement or career development or career trajectories)
MEDLINE (ProQuest)	(autis* OR asperger* OR ASD OR "autistic employees" OR "autistic workers") AND ("workplace accommodations" OR "job accommodations" OR "reasonable adjustments" OR "workplace support*" OR "occupational adjustment*" OR "inclusive employment") AND ("employment outcome*" OR employ* OR "job satisfaction" OR "employee well-being" OR retention OR performance OR "career progression")
PsycINFO	(DE "Autism Spectrum Disorders" OR autis* OR asperger* OR ASD OR "autistic employees" OR "autistic workers") AND ("workplace accommodations" OR "job accommodations" OR "reasonable adjustments" OR "workplace support*" OR "occupational adjustment*" OR "inclusive employment") AND ("employment outcome*" OR employ* OR "job satisfaction" OR "employee well-being" OR retention OR performance OR "career progression")

Eligibility Criteria

Inclusion and exclusion criteria were determined utilizing the Population-Intervention-Outcome (PIO) framework.

Population (P): Adults (≥16 years) with a clinical ASD diagnosis or self-identification as autistic, working in workplace or corporate environments.

Intervention (I): Any form of workplace accommodation, including assistive technology, sensory or environmental modifications, organizational or flexible work arrangements, job coaching, supervisory or relational support, and psychosocial interventions.

Outcome (O): Work-related outcomes such as job acquisition, retention, satisfaction, inclusion, performance, productivity, or workplace well-being.

The selection criteria for inclusion and exclusion are presented in Table 2.

**Table 2:** Selection criteria

Inclusion criteria	Exclusion criteria
The inclusion criteria include peer-reviewed publications written in English and published between 2010 and 2025. Study designs such as randomized controlled trials (RCTs), cohort studies, cross-sectional surveys, and qualitative designs. The study should focus on outcomes that are directly related to workplace functioning or employment experience. The study should only include publications written in the English language.	Studies that focus on children, adolescents, or education placements are excluded. Interventions that do not pertain to workplace accommodations, such as clinical therapy, are not eligible for inclusion. These may include non-peer-reviewed literature, editorials, or conference abstracts. Studies that involve a variety of neurodiverse groups but lack a specific differential analysis for employees with autism are also excluded.

Study Selection

**Figure 1:**
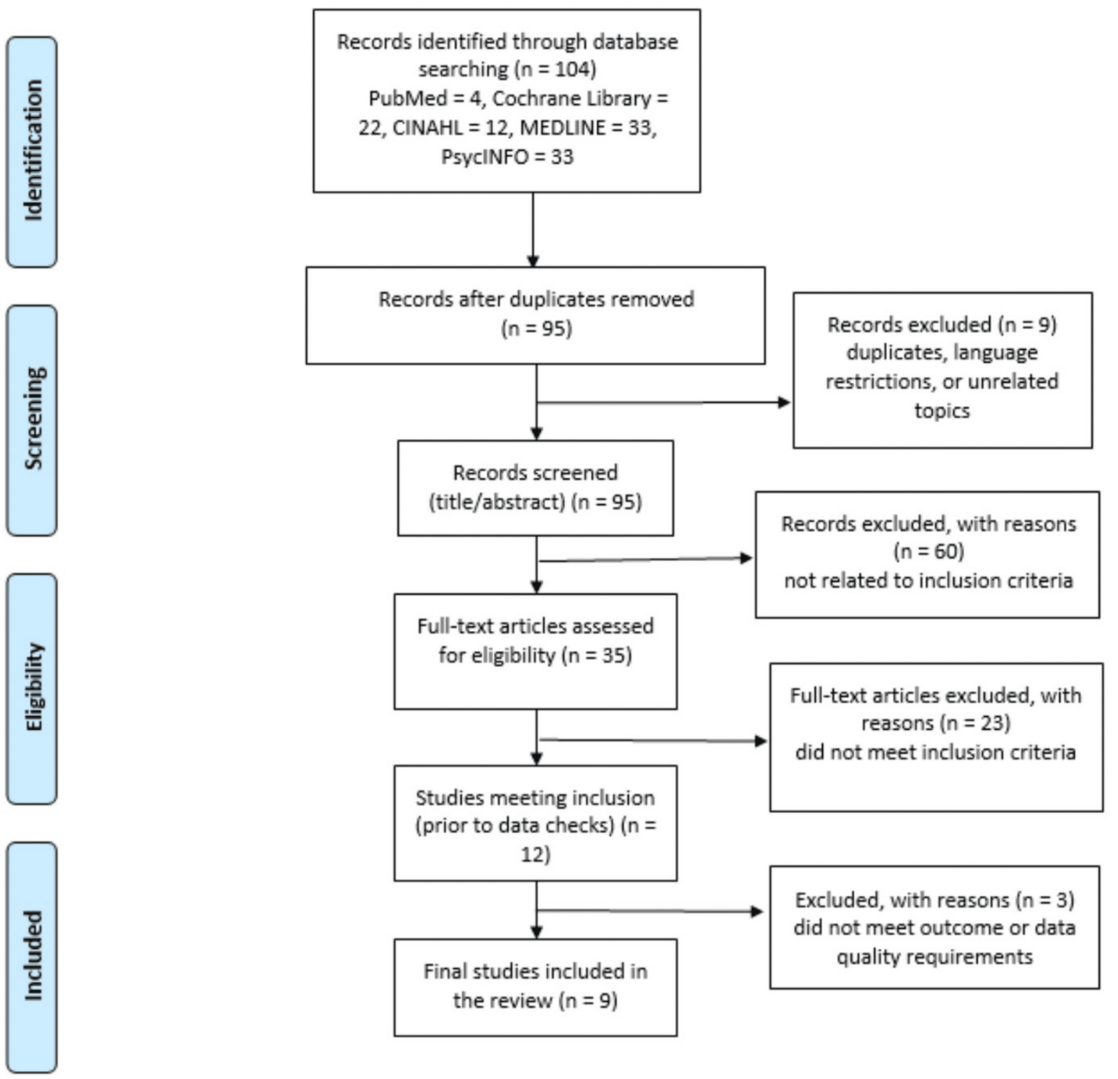
PRISMA flow diagram PRISMA: Preferred Reporting Items for Systematic Reviews and Meta-Analyses; MEDLINE: Medical Literature Analysis and Retrieval System Online; CINAHL: Cumulative Index to Nursing and Allied Health Literature

The PRISMA 2020 flow diagram illustrates the selection process and records reasons for exclusion at each step in order to maintain transparency (Figure 1).

Data Extraction

A pilot-tested standard data extraction form was constructed prior to full execution, and ten studies were selected (Table 3). The form recorded bibliographic details (author, year, country); study design and methodology; participant characteristics (sample size, age, gender distribution, diagnostic confirmation); industry sector or workplace setting; nature of workplace accommodations or interventions implemented; employment outcome measured (e.g., retention, job satisfaction, inclusion, performance); and key findings, including effect sizes and levels of significance where noted. Two reviewers independently extracted information, cross-checking to verify accuracy. Discrepancies were resolved by consensus. This systematic strategy allowed the mapping of evidence in four domains of support: assistive technology (e.g., adaptive computer software, communication aids); environmental and sensory accommodations (e.g., quiet rooms, lighting); organizational and supervisory supports (e.g., flexible working hours, line manager training); and skills-based and psychosocial interventions (e.g., job coaching, resilience programs). This classification allowed comparison of findings across varied study designs and outcome measures. The final ten studies included are mentioned in Table 3 (the study by Petty and Romualdez et al. was not considered for the final review) [46,69-79].

**Table 3:** Included studies

Ref no.	Author (Year)	Full reference
[46]	Stratton et al. (2023)	Stratton, E., Glozier, N., Woolard, A., et al. (2023). Understanding the vocational functioning of autistic employees: the role of disability and mental health. Disability and Rehabilitation, 45(9), 1508–1516.
[69]	Scott et al. (2018)	Scott, M., Falkmer, M., Girdler, S., Falkmer, T., & Bölte, S. (2018). Evaluating the effectiveness of an autism-specific workplace tool for employers: a randomised controlled trial. Journal of Autism and Developmental Disorders, 48(10), 3377–3392.
[70]	Harvey et al. (2021)	Harvey, M., Froude, E. H., Foley, K. R., Trollor, J. N., & Arnold, S. R. (2021). Employment profiles of autistic adults in Australia. Autism Research, 14(10), 2061–2077.
[71]	Wei et al. (2018)	Wei, X., Yu, J. W., Wagner, M., Hudson, L., Roux, A. M., Shattuck, P., & Blackorby, J. (2018). Job searching, job duration, and job loss among young adults with autism spectrum disorder. Journal of Vocational Rehabilitation, 48(1), 1–10.
[72]	Yon-Hernández et al. (2025)	Yon-Hernández, J. A., Gonzales, C., Bothra, S., et al. (2025). Early employment outcomes in autistic and non-autistic youth: challenges and opportunities. Journal of Autism and Developmental Disorders, 1–16.
[73]	Lee et al. (2024)	Lee, N. R., McQuaid, G. A., Grosman, H. E., Jayaram, S., & Wallace, G. L. (2024). Vocational outcomes in ASD: an examination of work readiness skills as well as barriers and facilitators to employment identified by autistic adults. Journal of Autism and Developmental Disorders, 54(2), 477–490.
[75]	Zalewska et al. (2016)	Zalewska, A., Migliore, A., & Butterworth, J. (2016). Self-determination, social skills, job search, and transportation: is there a relationship with employment of young adults with autism? Journal of Vocational Rehabilitation, 45(3), 225–239.
[77]	Martin et al. (2023)	Martin, V., Flanagan, T. D., Vogus, T. J., & Chênevert, D. (2023). Sustainable employment depends on quality relationships between supervisors and their employees on the autism spectrum. Disability and Rehabilitation, 45(11), 1784–1795.
[78]	Richardson et al. (2019)	Richardson, L., McCoy, A., & McNaughton, D. (2019). “He’s worth the extra work”: The employment experiences of adults with ASD who use augmentative and alternative communication (AAC) as reported by adults with ASD, family members, and employers. Work, 62(2), 205–219.
[79]	Katz et al. (2015)	Katz, N., Dejak, I., & Gal, E. (2015). Work performance evaluation and QoL of adults with high functioning autism spectrum disorders (HFASD). Work, 51(4), 887–892.

Quality Appraisal

All included studies were thoroughly assessed for quality using appropriate appraisal tools for their design: the Risk-of-Bias (RoB) 2.0 tool for randomized trials [64]; the Critical Appraisal Skills Programme (CASP) Cohort and Qualitative Checklists for longitudinal and interview studies [65,66]; and the Joanna Briggs Institute (JBI) critical appraisal checklists for cross-sectional surveys and descriptive studies [67]. Quality appraisal was performed by two reviewers independently. Overall, the majority of studies were classified as moderate to high quality with a relatively low risk of bias in sampling and in reporting (Table 4). Typical methodological strengths were an expression of clarity in research aims and alignment with workplace outcomes. However, some limitations were observed: the small sample size, the use of self-reported outcomes, the failure to randomize among non-RCTs, and the absence of follow-up for more than a year. We considered these factors during the synthesis process to avoid overgeneralizing the results.

**Table 4:** Quality appraisal of included studies Review Criteria Score (1-3): High Quality (3 points) = Meets most criteria, low risk of bias, strong methodology; Moderate Quality (2 points) = Some limitations, potential bias, but still useful; Low Quality (1 point) = Major limitations, unclear reporting, weak evidence. WHODAS: World Health Organization Disability Assessment Schedule; DASS-21: Depression Anxiety Stress Scales-21; RCT: randomized controlled trial; RoB 2: Revised Cochrane Risk-of-Bias Tool for Randomized Trials; SPARK: Simons Foundation Powering Autism Research for Knowledge cohort; AQ: autism quotient; ADOS: Autism Diagnostic Observation Schedule; CASP: Critical Appraisal Skills Programme; JBI: Joanna Briggs Institute; ROBINS-I: Risk of Bias in Non-Randomized Studies of Interventions; WPE: workplace evaluation; QoL: quality of life; LMX: leader–member exchange; AAC: augmentative and alternative communication; Dx: diagnosis; STROBE: Strengthening the Reporting of Observational Studies in Epidemiology. Note. STROBE was used only as a reporting-transparency checklist and not as a risk-of-bias or methodological quality tool. Numerical STROBE item counts were removed. Overall quality ratings represent narrative appraisals based on study design, methodological clarity, and potential bias.

Ref	Reference	Tool used	Bias risk	Strengths	Limitations	Overall quality	Tool score	Review criteria score (1–3)
1	Stratton et al., 2023 (Disability & Rehabilitation) [46]	STROBE (cross-sectional)	Moderate	Clinical Dx confirmation; validated instruments (WHODAS, DASS-21); focused vocational outcomes	Young sample (M≈23); employment sectors not detailed; cross-sectional; potential clinic sample bias	Moderate	~8/12 items	2
2	Scott et al., 2018 (JADD) [69]	RoB 2	Low–moderate (randomization described; self-reported outcomes; usage heterogeneity)	RCT design; preregistered style methods; usable, real-world employer sample; clear primary/secondary outcomes	No blinded outcomes; employer self-report; underpowered for between-group effects; short follow-up; contamination via DES support possible	Moderate–High	~8/10 items met	3
3	Wei et al., 2018 (JVR) [71]	STROBE (cohort)	Low–moderate (survey weighting; some missing data; listwise deletion)	Nationally representative dataset; rigorous weighting; clear comparative analyses, and multiple predictors	Self-/parent-report; older data (2009); education-based Dx; no employer verification of job loss	Moderate–High	~10/22 cohort items	3
4	Yon-Hernández et al., 2025 (JADD) [72]	STROBE (cohort)	Moderate (good measures; modest sample; potential residual confounding)	Comparison group; validated Dx (ADOS-2); multivariable models; clear effect sizes	Convenience sub-sample; sector spread unequal; limited power for subgroup analyses; early adulthood only	Moderate–High	~9/22 STROBE items well reported (key cohort items)	3
5	Lee et al., 2024 (JADD) [73]	STROBE (cross-sectional)	Moderate (self-report; SPARK volunteer bias)	Large n; validated screen (AQ-28); pre-specified models; practical barriers/facilitators identified	Cross-sectional; diagnosis confirmation indirect; sector data sparse; female-skewed sample may limit external validity	Moderate	~8/12 items	2
6	Zalewska et al., 2016 (JVR) [75]	STROBE (cross-sectional/associational)	Moderate	Large, weighted sample; operationalized constructs; practical, policy-relevant findings (transportation)	Partial scales (self-determination subset); temporal mismatch of measures; listwise deletion; correlational only	Moderate	~7/12 items	2
7	Harvey et al., 2021 (Autism Research) [70]	STROBE (cross-sectional)	Moderate (self-selection; self-report; some self-ID diagnoses)	National scope; mixed-methods adds depth; detailed adjustments/utilization analyses; appropriate stats	Non-probability sample; cross-sectional (no causality); under-representation of managers; self-reported Dx for some	Moderate	~7/12 core STROBE items clearly met	2
8	Martin et al., 2023 (Disability & Rehabilitation) [77]	CASP Qualitative	Low–moderate	Triangulated perspectives (employees, managers, coaches); rich thematic analysis; theory-linked (LMX)	Small n, purposive sampling; sector heterogeneity limits transferability; researcher reflexivity reporting is limited	Moderate	~8/10 CASP prompts adequately addressed	2
9	Richardson et al., 2019 (Work) [78]	CASP Qualitative	Low–moderate	Under-studied population (ASD+AAC); multi-stakeholder triangulation; clear themes on accommodations/barriers	Small purposive sample; transferability limits; AAC type and severity heterogeneity; limited reflexivity detail	Moderate	~8/10 CASP prompts	2
10	Katz et al., 2015 (Work) [79]	JBI Cohort Checklist / ROBINS-I (non-randomized)	Moderate–high (no control; small n)	Prospective follow-up with multiple timepoints; objective/team WPE plus self-QoL; 100% retention	No comparator; selection bias; limited demographics; short 9-month horizon; program context may limit generalizability	Moderate	~6/11 JBI items	2

Data Synthesis

Due to heterogeneity in interventions, outcomes, and study designs, a meta-analysis was not feasible. Instead, a narrative synthesis was undertaken, following guidance as outlined for the synthesis of diverse forms of evidence [68]. Quantitative results were described in terms of effect direction, strength, and statistical significance, whereas qualitative results were synthesized thematically. The results were synthesized across the four themes of accommodations: assistive technology, environmental/sensory, organizational/supervisory, and psychosocial/skill-based. This design provided both an evidence map of intervention types and a more nuanced perspective on how accommodations influence employment outcomes such as retention, satisfaction, and workplace inclusion.

Ethical Considerations

The review of secondary data from published studies did not need ethical approval. However, aspects of responsible research practice, accurate representation, respectful citations, and engaging critique with autistic voices were upheld throughout.

Results

Overview of Included Studies

During the review period 2010-2025, 10 studies met the inclusion criteria: one randomized controlled trial [69], three cohort studies [70-72], four cross-sectional or survey-based studies [73-76], and two qualitative multi-informant studies [77,78] (Table 5). In combination, these designs ensured triangulation between experimental, observational, and experiential data relevant to workplace accommodations and employment outcomes. The studies were overwhelmingly based in Anglophone settings, such as Australia [69,70,75], the United States [71,73,76], Canada [77], and Israel [79], with one comparative international study of autistic and non-autistic adolescents [72]. Publications were focused in the past decade (2015-2025) to capture the most contemporary evidence reflecting recent diagnostic updates, policy shifts, and evolving neurodiversity-affirming employment practices, mirroring increased focus on employment participation among autistic adults and youth in transition.

**Table 5:** Study characteristics P: population; I: intervention/exposure; O: outcome; RCT: randomized controlled trial; NLTS2: National Longitudinal Transition Study-2; AAC: augmentative and alternative communication; QoL: quality of life; HFASD: high-functioning autism spectrum disorder; IEST™: Integrated Employment Support Tool (autism-specific employer training/guidance).

Ref	Reference	Study type	Why include	Population (P)	Intervention (I)	Outcome (O)	Results
1	Stratton et al., 2023 [46]	Cross-sectional secondary analysis	Links mental health, support, and functioning.	Autistic employees	Workplace accommodations & supports	Functioning, retention	Poor mental health ↓ functioning; supports ↑ retention
2	Scott et al., 2018 [69]	RCT (2-arm)	Only workplace RCT; tests autism-specific employer tool	Employers of autistic adults	IEST™ tool (training + guidance for accommodations)	Employer knowledge, workplace inclusion	↑ Employer self-efficacy; autistic staff reported better inclusion
3	Harvey et al., 2021 [70]	Cross-sectional mixed-methods	Large national cohort; links support with outcomes	Autistic adults	Workplace supports/adjustments (exposure)	Employment status, satisfaction, retention	Supports linked with ↑ satisfaction & retention
4	Wei et al., 2018 [71]	Cohort (secondary analysis, NLTS2)	Young autistic adults	Transition supports, workplace accommodations	Job acquisition, duration, loss	Supports extended job duration; job loss high w/out accommodations	Accommodations linked to longer job duration and lower job loss; absence increased instability
5	Yon-Hernández et al., 2025 [72]	Cohort-sequential (from longitudinal study)	Early workplace outcomes with accommodations	Autistic youth/employees	Transition & workplace accommodations	Retention, satisfaction	Accommodations linked to improved satisfaction & retention
6	Lee et al., 2024 [73]	Cross-sectional online survey	Identifies workplace barriers/facilitators	Autistic adults	Work readiness & accommodations	Employment status, barriers	Barriers = social/sensory; facilitators = coaching & structure
7	Zalewska et al., 2016 [75]	Observational survey (NLTS2 secondary)	Functional skills predicting employment	Young autistic adults	Supports in social skills, job search, transport	Employment status	Self-determination & social skills = strongest predictors
8	Martin et al., 2023 [77]	Qualitative case study	Explores supervisor–employee relationships as accommodations	Employed autistic adults	Supervisor support, workplace adjustments	Retention, job satisfaction	Positive supervisor relationships → ↑ retention & satisfaction
9	Richardson et al., 2019 [78]	Qualitative (multi-informant interviews)	Focus on AAC users in workplaces	Autistic adults using AAC	AAC workplace supports	Retention, inclusion	AAC accommodations ↑ productivity & inclusion
10	Katz et al., 2015 [79]	Prospective non-randomized cohort (work-placement program)	Evaluates workplace performance and QoL	Adults with HFASD	Workplace accommodations (varied)	Work performance, quality of life	↑ Work performance correlated with ↑ QoL

Populations differed by study design and recruitment route. The RCT [69] recruited employers of autistic adults, whereas observational and cohort studies recruited autistic adults or youths without intellectual disabilities using national datasets [71,73,76], clinical or registry cohorts [70,75], or supported employment programs [77-79]. Sample sizes varied from small qualitative groups (N=18 in [77]; N=9 in [78]) to high-volume national surveys (N=660 in [71]; N=281 in [73]).

Industry sectors included retail, food service/hospitality, clerical/administrative work, manufacturing, information technology, healthcare, and professional jobs. Studies continually tested the effects of real-world accommodations, such as augmentative communication and assistive technology [78], organizational or environmental changes [70,75], supervisory and relational supports like job coaching and manager-employee relations [72,77], and skills-based or psychosocial supports [73,76,79].

The studies measured outcomes such as job attainment and search time [71,76], job stability and retention [70,79], job inclusion and satisfaction [77,78], and work performance and productivity [69,79]. Together, the body of evidence synthesizes employer insights, longitudinal trajectories, population-based rates, and lived experiences, providing an overarching picture of how workplace accommodations impact autistic individuals' employment outcomes.

Participant Characteristics

Across the 10 studies included in this report, participant samples differed significantly in size, age range, gender distribution, diagnostic criteria, and recruitment strategies (Table 6). Sample sizes spanned from tiny qualitative cohorts to national datasets of considerable size. The smallest sample was nine autistic adults who used augmentative and alternative communication (AAC) in [78], and the largest one was 660 autistic young adults in the National Longitudinal Transition Study 2 (NLTS2)-based cohort [71]. The other cohorts and surveys had between 88 and 281 autistic adults [73,75], while mid-sized observational studies had 26 to ~570 participants [76,79].

**Table 6:** Overview of participant characteristics for included studies N: total number of participants; n: number of participants by sex or subgroup; yrs: years; SD: standard deviation; Dx: diagnosis; FSIQ: Full-Scale Intelligence Quotient; HFASD/HFA: high-functioning autism spectrum disorder/high-functioning autism; ADOS-2: Autism Diagnostic Observation Schedule, Second Edition; AQ-28: Autism Spectrum Quotient (28-item version); AAC: augmentative and alternative communication; DSM-IV/DSM-IV-TR/DSM-5: Diagnostic and Statistical Manual of Mental Disorders (4th, 4th Text Revision, and 5th Editions); EMR: electronic medical record; WTAR: Wechsler Test of Adult Reading; WASI-II: Wechsler Abbreviated Scale of Intelligence, Second Edition; IDEA: Individuals with Disabilities Education Act; NLTS2: National Longitudinal Transition Study 2 (U.S. national dataset); SPARK: Simons Foundation Powering Autism Research Knowledge (online research registry); ALSA: Australian Longitudinal Study of Autistic Adults; PSE: postsecondary education; DASS-21: Depression, Anxiety, and Stress Scales (21-item version); ABS: Australian Bureau of Statistics; USA: United States of America; n.r.: not reported; %: percentage; M: male; F: female; and Other: gender diverse or non-binary participants.

Author (year)	N (n = sex/gender)	Mean age (SD), range	% Clinical diagnosis of autism	Mean age at diagnosis (SD)	Mean FSIQ (SD); measure	% Ethnic group/race	% Educational attainment	% Comorbidities	Location; recruitment methods
Stratton et al. (2023) [46]	88 autistic adults (65M, 23F)	23.3 (6.7), 16–41 yrs	100% Dx (DSM-IV-TR/DSM-5; ADOS-2 ≥7; IQ ≥70 WTAR)	n.r.	Mean IQ ≥70; WTAR	n.r.	n.r.	Depression is common, measured with DASS-21	Australia; clinical cohort sample
Scott et al. (2018) [69]	84 employers of autistic adults (not autistic participants themselves)	Employees ≥18 yrs (range 15–64, working-age adults, ABS definition)	100% (self-ID as Asperger’s, HFA, autism; DSM-IV criteria)	n.r.	n.r.	n.r.	n.r.	n.r.	Australia; employers recruited via the workplace and Disability Employment Services
Harvey et al. (2021) [70]	149 autistic adults (60 M, 82 F, 7 Other)	41.0 (10.8), 25–80 yrs	86% formal Dx; 14% self-identifying	n.r.	n.r.	Majority White (Australia); breakdown n.r.	88% postsecondary education	n.r.	Australia; ALSAA national survey, self-report
Wei et al. (2018) [71]	660 autistic young adults (84% M, 16% F)	21–25 yrs	School classification “autism” (≥95% meet DSM-IV criteria)	n.r.	n.r.	Diverse; Black/Hispanic under-represented; subgroup analysis reported	Postsecondary enrollment: 30–40%	n.r.	USA; NLTS2 longitudinal survey (nationally representative)
Yon-Hernández et al. (2025) [72]	51 autistic (40M, 11F), 48 non-autistic (37M, 11F)	18–23 yrs	100% (ADOS-2, IQ ≥70 WASI-II)	n.r.	Mean IQ ≥70 (WASI-II)	n.r.	Post-secondary enrollment recorded; rates not reported	Executive function difficulties are common	USA; cohort-sequential study, longitudinal follow-up
Lee et al. (2024) [73]	281 autistic young adults, majority female at birth	22–39 yrs	94% screened positive AQ-28; ≥98% confirmed Dx in EMR (SPARK validation)	n.r.	n.r.	Predominantly White, non-Hispanic	n.r.	n.r.	USA; SPARK online registry, Research Match
Zalewska et al. (2016) [75]	~570 autistic young adults (84% M, 16% F)	22–25 yrs	School classification “autism” (IDEA category)	n.r.	n.r.	Diverse (from NLTS2)	Postsecondary education variable	n.r.	USA; NLTS2 Wave 5 secondary analysis
Martin et al. (2023) [77]	18 participants (5 autistic employees, 8 managers, 5 job coaches); autistic = 8M, 1F	Adults, young-to-middle age; range n.r.	100% documented autism diagnosis	n.r.	n.r.	n.r.	n.r.	n.r.	Canada; supported employment program case studies
Richardson et al. (2019) [78]	9 autistic adults using AAC (sex/gender n.r.); +10 family, 8 employers	Adults (post-school age); range n.r.	100% Dx + AAC users	n.r.	n.r.	n.r.	n.r.	n.r.	USA; purposive sample, qualitative interviews
Katz et al. (2015) [79]	26 adults with HFASD; sex not specified	18–40 yrs	100% HFASD (clinical Dx)	n.r.	IQ > 70 implied	n.r.	n.r.	n.r.	Israel; recruited into a structured placement program

With regard to age, a majority of the studies targeted working-aged adults (18-64 years). A study [69] targeted employers but included employees ≥18 years. A wide adult age range (25-80 years, mean ≈ 41 years) was found in [70], and [79] investigated young to middle-aged adults (18-40 years); [75] had a younger employed population (M=23.3 years), and [73] sampled young adults (22-39 years old). We provided transition-age youth samples in [71,72,76], which captured the crucial school-to-work transition period.

Gender reporting differed significantly [70]; it included women with 55% of the sample and men at 40%, while 5% reported otherwise. Two studies [75,76] had disproportionately male samples (74% and ~84% male, respectively). Large NLTS2 datasets [71,76] also contained this male dominance. Conversely, [73] identified their registry-based sample as being largely assigned female at birth, which is consistent with published female over-representation in online autism registries [78,79], and did not provide participant gender consistently, making it less comprehensible.

To our knowledge, most studies used formal clinical diagnoses of ASDs, occasionally validated by standardized assessment methods like the Autism Diagnostic Observation Schedule (ADOS-2) [72,75]. In [73], diagnoses were verified with electronic medical records for close to all registry participants. On the other hand, one study had a percentage of self-identifying autistic adults (≈14%) as well as those with formal diagnoses [70]. Two studies [71,76] used special education classifications of autism from school records, which surveillance studies indicate align with Diagnostic and Statistical Manual of Mental Disorders (DSM)-IV/DSM-5 criteria in over 95% of cases [79]. They required a diagnosis of high-functioning autism spectrum disorder (HFASD), and [78] focused on individuals with both autism and AAC use, also based on recorded clinical diagnoses.

Recruitment practices mirrored the study design. Nationally representative datasets like NLTS2 [71,76] offered weighted, population-level data, although diagnoses were based on educational classification, and some measures were outdated. Registry and survey research studies [70,73] employed volunteer and self-report data, thus possibly biasing toward highly educated or more involved participants. Clinical samples [75] provided a standardized diagnosis, but they could under-represent community experiences more broadly. Employer- or program-based samples [69,77,78] preserved ecological validity but tended to be small and context-specific. Lastly, purposive qualitative sampling [78] permitted insights into exceptional populations (AAC users) but restricted generalizability.

Study Quality Appraisal

We assessed the methodological quality of the included studies using well-established critical appraisal tools specific to study design (Table 4). In particular, the RoB 2 tool was used in the randomized controlled trial [69], Strengthening the Reporting of Observational Studies in Epidemiology (STROBE) checklists in cross-sectional and cohort analyses [70-73,75,76], the JBI cohort checklist in non-randomized prospective data [79], and the CASP Qualitative Checklist in interview-based studies [77,78]. These tools offered a systematic assessment of internal validity, reporting transparency, and biases.

Patterns of Risk of Bias

On balance, the majority of studies were assessed as moderate in quality, with two close to achieving higher quality levels [69,71]. The RCT had strengths in preregistration and well-defined outcome measures, but had a risk of bias through employer self-report, absence of blinding, and low statistical power for between-group effects. Cohort analyses employing the NLTS2 database [71,76] were advantaged by representative sampling and strong analytic weighting, albeit with outcome data based on self- or parent-reporting and diagnostic classification based on education. Cross-sectional surveys [70,73,75] provided substantial sample sizes and validated screening tools; nevertheless, self-selection bias, reliance on self-reporting, and the lack of causal inference hindered interpretation. Qualitative research [77,78] was deemed robust in terms of triangulation and theme coherence; however, it was constrained by small purposive samples and incomplete reflexivity reporting [79]. The prospective cohort provided objective performance ratings at multiple timepoints, but the absence of a comparator group and sparse demographics limited its external validity.

Methodological Gaps

Some typical limitations were found across the evidence base. First, small sample sizes were common in intervention and qualitative work, limiting generalizability and subgroup analysis. Second, self-report use was present across both quantitative and qualitative designs, with issues around recall bias, social desirability, and non-reporting of unmet needs or failed employment experiences. Third, the demographic reporting was incomplete, especially concerning gender diversity, comorbid conditions, and socio-economic background, preventing an understanding of intersectional experiences. Finally, most studies were short-term, with few tracking employment trajectories beyond one or two years, thus under-representing long-term stability and career progression.

Types of Workplace Accommodations Identified

The 10 studies summarized here identify a wide variety of workplace accommodations, ranging from assistive technology to environmental and organizational changes, supervisory and relational support, and skill-based or psychosocial interventions (Table 7). No single approach was used across all cases, but similar patterns were identified regarding the types of support most frequently linked to successful employment outcomes.

**Table 7:** Comparative evidence strength of assistive, environmental, supervisory, and skills-based supports Ref: study reference number as cited in the systematic review; RCT: randomized controlled trial; QoL: quality of life; OR: odds ratio; p: probability value; d: Cohen’s effect size; ID: intellectual disability; WHODAS 2.0: World Health Organization Disability Assessment Schedule, 2nd Edition; DASS-21: Depression, Anxiety and Stress Scales, 21-item version; AWSQ: Autism Work Skills Questionnaire; SPARK: Simons Foundation Powering Autism Research Knowledge (online research registry); NLTS2: National Longitudinal Transition Study 2 (U.S. national dataset); HFASD: high-functioning autism spectrum disorder; AAC: augmentative and alternative communication; IEST™: Integrated Employment Success Tool; FT: full-time employment; P: population; I: intervention; O: outcome.

Group	Ref	Study design	Year	Population (P)	Intervention (I)	Outcome (O)	Results
Assistive Technology	Richardson et al., 2019 [78]	Qualitative interviews (multi-stakeholder)	2019	9 autistic adults using AAC, 10 family members, 8 employers	AAC device use at the workplace: flexible communication methods	Employment retention, satisfaction, and inclusion	Employment is feasible but requires extra effort; facilitators = supportive supervisors and job coaches; barriers = stigma, limited communication training.
Environmental/Sensory & Organizational	Zalewska et al., 2016 [75]	Observational survey (NLTS2)	2016	~570 autistic young adults (US, 22–25 yrs)	Transport independence (driver’s license, independent travel)	Employment (ever worked, current job, tenure)	Transport independence strongly predicted employment (driver’s license OR=5.2; independent travel OR=4.8).
	Harvey et al., 2021 [70]	Cross-sectional mixed-methods	2021	149 autistic adults (Australia)	Workplace adjustments: sensory modifications, written instructions, flexible hours, remote work, altered workflows	Employment status, utilization, and underemployment	25% received adjustments; adjustments ↑ odds of appropriate employment (OR=3.14, p=0.035). Fear of disclosure limited uptake.
Supervisory/Relational Supports	Scott et al., 2018 [69]	RCT	2018	84 employers of autistic adults	Integrated Employment Success Tool (IEST™): 8-module autism-specific guide for managers	Employer self-efficacy, attitudes toward autistic workers	↑ Employer self-efficacy (p=0.016, d=0.58); attitudes improved with regular IEST use.
	Martin et al., 2023 [77]	Qualitative case study	2023	5 autistic employees, 8 managers, 5 job coaches	Supported employment + job coaches mediating manager–employee relationship	Job retention, satisfaction, and workplace climate	High-quality manager–employee relationships led to long-term employment, pride, and team cohesion. Poor relationships → contract terminations.
Skills & Psychosocial Supports	Stratton et al., 2023 [46]	Cross-sectional cohort	2023	88 employed autistic adults (no ID)	Mental health & disability (WHODAS 2.0, DASS-21)	Vocational disability, disability days	Disability & depression significantly predicted poor vocational functioning (OR=1.08, p=0.035).
	Wei et al., 2018 [71]	Cohort study (NLTS2)	2018	660 autistic young adults (21–25 yrs)	Predictors: conversation ability, functional skills, postsecondary education	Job search length, tenure, and firing	Autistic youth had longer job searches (14 mo), the lowest self-finding rate (22%), but longer job tenure (24 mo). Conversational skills ↑ job seeking & tenure.
	Yon-Hernández et al., 2025 [72]	Cohort-sequential	2025	51 autistic vs 48 non-autistic youth (18–23 yrs)	Employment supports: job coaching, supervisor assistance, group classes	Employment rate, job type, duration	Autistic youth had lower-paid employment (50% vs. 78%) and fewer FT roles (3% vs 17%). Post-secondary education strongly predicted employment (OR=5.97, p=0.003).
	Lee et al., 2024 [73]	Cross-sectional survey	2024	281 autistic young adults (SPARK, USA)	Work readiness skills (AWSQ)	Employment status, hours worked	Work style/adaptability predicted independence (model accuracy ~70%). Barriers = burnout, poor fit; facilitators = training, employer education.
	Katz et al., 2015 [79]	Prospective cohort (9-month follow-up)	2015	26 adults with HFASD (18–40 yrs)	Structured work placement program: tailored job matching + support	Job retention, work performance, QoL	100% retention; performance stable-high; significant ↑ QoL (esp. self-competency, p<0.05).

Assistive Technology

Assistive technology was raised specifically in one qualitative study with autistic adults who were dependent on AAC devices [78]. Participants, families, and employers explained how AAC tools like speech-generating devices, communication boards, or adapted software helped autistic employees to accomplish core work tasks, contribute during meetings, and remain employed (Table 7). Yet the research also highlighted that effective implementation relied upon supervisors' flexibility, regular job coaching, and workplace training in how to communicate with AAC users. Relational support alone, without these, made technology ineffective. Stigma, inadequate coworker training, and variable employer commitment were barriers that could deplete inclusion and productivity. Though the evidence base was restricted to a purposive sample of modest toddlers, the findings emphasize that assistive technology is a possible but costly strategy for accommodation. An environment that recognizes and values diverse communication needs is more important for its efficacy than the device itself.

Environmental/Sensory and Organizational Accommodations

A number of studies document organizational and environmental changes to limit obstacles facing autistic workers [70,75]. The types of adjustments shared were flexible working time, provision for homework, written instructions for tasks, workflow modifications, and sensory changes such as quiet environments or limited noise exposure. In Harvey et al.’s national cohort, only 25% of autistic adults reported receiving such adjustments, yet logistic regression analyses indicated that accommodations significantly increased the likelihood of appropriate employment (OR=3.14, p=0.035). Despite this, many participants expressed a desire for further support, particularly in relation to sensory sensitivities and managerial understanding of autism, with fear of disclosure often acting as a barrier to requesting adjustments (Table 7).

Transportation independence was also a key organizational variable. Evaluations of the NLTS2 cohort [75] showed that having a driver’s license (OR=5.2) or being able to maneuver around independently (OR=4.8) was a powerful predictor of employment. These results demonstrate that organizational accommodations extend beyond the specific work environment, encompassing systemic supports such as accessible transportation. Together, research indicates that although environmental and organizational adjustments can significantly enhance access to and sustainability of jobs, uptake is still patchy, and disclosure issues continue to restrict provision.

Supervisory/Relational Supports

A recurring theme throughout the research was the significance of supervisory and relational adjustments. The single RCT in the review [69] tested the Integrated Employment Success Tool (IEST™), a manual for employers with modules on job design, interviews, and workplace support. Frequent users of IEST™ showed significant gains in self-efficacy (p=0.016, d=0.58) and improved attitudes toward autistic staff. The findings suggest that employer training can change workplace culture and improve inclusion (Table 7).

Qualitative case studies supported this view. Martin et al. [77] showed that good-quality manager-employee relationships, facilitated by job coaches, were core to long-term employment. When managers communicated, had mutual understanding, and exchanged ongoing feedback, autistic staff kept positions in the long term, felt pride, and contributed to healthy team climates. Conversely, unsupported or low-quality relationships between managers and employees often led to the termination of contracts, even when employees had the necessary skills. In the same vein, Richardson et al. [78] identified how supervisors' open-mindedness to task adjustments and working together with AAC users enabled inclusion.

Skills and Psychosocial Supports

Several studies established the contribution of skills-based and psychosocial support towards encouraging employment engagement (Table 7). Katz et al. [79] observed that a goal-planned work placement program with individualized job matching and repeated professional support had 100% retention at nine-month follow-up, along with enhanced quality of life, especially self-competence (p < 0.05). In the same vein, Lee et al. [73] demonstrated that vocational independence was predicted by work style and adaptability skills, such that regression models correctly classified 70-77% of outcomes. Functional skills and conversational ability also predicted job tenure and independent job-finding, according to longitudinal analyses [71].

However, the research also highlighted obstacles, such as burnout, poor job fit, and diminished readiness training. Stratton et al. [46] further noted that depression and disability days significantly lowered vocational functioning. In combination, the findings place psychosocial and skill-based interventions, specifically those involving adaptability, communication, and readiness, as high-value strategies for maintaining meaningful employment.

Employment Outcomes Reported

The included studies reported a wide range of employment-related outcomes, clustered around job acquisition and search duration, retention and stability, job satisfaction and inclusion, and work performance or productivity. Collectively, these outcomes demonstrate both the barriers autistic employees face and the benefits of tailored accommodations (Table 8).

**Table 8:** Employment outcomes summary Ref: reference number as cited in the systematic review; DSM-IV/DSM-IV-TR/DSM-5: Diagnostic and Statistical Manual of Mental Disorders (4th, 4th Text Revision, and 5th Editions); ASD: autism spectrum disorder; HFA/HFASD: high-functioning autism/high-functioning autism spectrum disorder; ADOS-2: Autism Diagnostic Observation Schedule, Second Edition; WASI-II: Wechsler Abbreviated Scale of Intelligence, Second Edition; WTAR: Wechsler Test of Adult Reading; AQ-S: Autism Spectrum Quotient (Short Form); SPARK: Simons Foundation Powering Autism Research Knowledge Registry; IDEA: Individuals with Disabilities Education Act; NLTS2: National Longitudinal Transition Study 2 (U.S.); ANZSCO: Australian and New Zealand Standard Classification of Occupations; DES: Disability Employment Service; IEST™: Integrated Employment Success Tool; AAC: augmentative and alternative communication; WPE: work performance evaluation; QoL: quality of life; LMX: leader–member exchange theory; WHODAS 2.0: World Health Organization Disability Assessment Schedule (2nd Edition); DASS-21: Depression, Anxiety and Stress Scales (21-item version); AWSQ: Autism Work Skills Questionnaire; OR: odds ratio; p: probability value; B: unstandardized beta coefficient; V: Cramér’s V (effect size for association); d: Cohen’s effect size; FT: full-time; PT: part-time; NR/n.r.: not reported.

Ref	Reference	Diagnosis criteria	Employment sector	Workplace accommodations	Employment outcomes	Key findings/effect sizes
1	Stratton et al., 2023 (Disability & Rehabilitation) [46]	Autism diagnosis confirmed via DSM-IV-TR or DSM-5, ADOS-2 cutoff ≥ 7, IQ ≥70 (WTAR)	Mixed (part-time = 68%, full-time = 32%); sectors not specified	No intervention was studied; assessed disability (WHODAS 2.0) and mental health (DASS-21). Focus: relationship between disability, depression/anxiety/stress, and vocational functioning	47% reported ≥1 disability day in the prior month (mean 5.3 days, range 1–30). Vocational disability median score = 37.5/100 (moderate). No effect of IQ or autism severity.	Higher overall disability and depression predicted both disability days (Disability OR=1.06, p=0.009; Depression OR=1.08, p=0.035) and vocational disability (Disability B=1.18, p<0.001; Depression B=1.01, p=0.049). Anxiety/stress is not significant in multivariable models. Effect sizes: large for disability days (d=0.94), and depression (d=0.80). Communication, social, and participation domains are the most impaired.
2	Scott et al., 2018 (JADD) [69]	Employees self-identified as Asperger’s, HFA, or autism; DSM-IV ASD criteria (APA, 2000)	Broad sectors: manufacturing (15.5%), healthcare/social assistance (13.1%), finance/insurance (7.1%), others representative of the Australian workforce	Intervention: Integrated Employment Success Tool (IEST™) – autism-specific manual with 8 modules, checklists, and video tutorials to guide job advertising, interviews, commencement, modifications, and ongoing support. Control: usual care via Disability Employment Service (DES) providers	Primary outcome: Employer self-efficacy (ESES). Secondary: Attitudes (SATWD).	Significant within-group improvement in self-efficacy for intervention (p=0.016; medium effect d=0.58). No significant between-group differences in self-efficacy or attitudes. Regular IEST™ users (weekly/fortnightly) showed significant gains in both self-efficacy (p=0.018) and attitudes (p=0.018).
3	Harvey et al., 2021 (Autism Research) [70]	Formal diagnosis (ASD, Asperger’s, HFA, etc.) n=128; self-identifying autistic n=21; all completed AQ-S (97% > cutoff)	All 8 ANZSCO groups represented. Top groups: Professionals (40%), Clerical/Admin (17%), and Community/Personal Service (13%). Only 6% managers vs. 12% gen. workforce	25% received adjustments. Types: supportive environments, sensory modifications, written instructions, flexible hours, remote work, and altered workflows. 34% desired adjustments (esp. disability understanding, sensory supports, and flexibility). Fear of disclosure is common.	86% employed (n=128), 14% unemployed (n=21). 37% underemployed (by hours or skill). 51% underutilized (vs. 14% of the national workforce). Mean weekly hours: 29.1 (FT: 54%, PT: 46%). High education (88% postsecondary) but mismatch with jobs (only 47% at skill level 1, though 74% qualified at level 1).	Logistic regressions: More autistic traits ↑ odds of appropriate utilization (OR=1.89 per 10 AQ-S points, p=0.003) and employment (OR=1.77, p=0.018). More social supports ↑ utilization (OR=1.34, p=0.015). Workplace adjustments ↑ appropriate employment (OR=3.14, p=0.035). Unemployment is 3x higher than the national rate.
4	Wei et al., 2018 (JVR) [71]	School special education classification “autism” (≥95% meet DSM-IV criteria in surveillance studies)	Varied (office/admin, food service, transportation, material moving, etc.); jobs mostly low-wage, part-time	No experimental intervention; predictors included demographic (age, gender, race, income), conversation ability, functional cognitive skills, and postsecondary education enrollment	<30% actively job searching at interview; only 22% found job on their own (lowest of 5 disability groups); mean job search 14 months (vs. 2–12 months in peers); average job duration 23.5 months (longer than LD, SLI, ED; similar to MR); job loss mostly due to temporary jobs ending (50%) vs quitting (20%) or firing (11%).	Challenges: ASD group was less likely to seek jobs (29% vs 49–60% in LD/SLI/ED), took significantly longer to find work, and had the lowest independent job-finding rate. Strengths: Once employed, autistic young adults held jobs longer (24 months vs 16 months for general peers) and reported high satisfaction (≈90% liked jobs and were treated well). Predictors: Better conversational ability → ↑ job seeking and duration; higher functional skills → ↑ odds of independent job finding (OR=1.21, p<0.01 postsecondary education odds of self-finding a job but shorter tenure. Higher-income households search for longer tenures. Males are more likely to be fired. Black youth and Hispanic individuals being at risks="">.
5	Yon-Hernández et al., 2025 (JADD) [72]	Autistic diagnosis confirmed via ADOS-2; IQ ≥70 (WASI-II)	Autistic concentrated in retail (52%); Non-autistic more diverse (food service 21%, sciences 12%, arts/media 12%, education 14%)	Autistic youth are more likely to receive job coaching (13%), group classes (7%), or supervisor assistance (3%); 77% worked independently vs 100% of non-autistic peers	Any work experience: 67% autistic vs 86% non-autistic; paid only: 50% vs 78%. Autistic individuals had fewer full-time roles (3% vs 17%), shorter job durations, and relied more on WAI (26%) or supported employment (16%) than competitive hiring (48% vs 95%).	Employment disparities persist. Executive function difficulties predicted a lower likelihood of employment (OR=1.069, p=.019); post-secondary education strongly predicted a higher likelihood (OR=5.97, p=.003). Cramér’s V values: job acquisition V=.50 (large), sector V=.55 (large), support V=.38 (moderate).
6	Lee et al., 2024 (JADD) [73]	Community ASD dx self-reported; SPARK cohort (≥ 98% dx confirmation in EMR in validation study); 94% screened positive on AQ-28 (>65).	Not the focus; sectors not reported (varied/NR).	No tested intervention; the study probed perceived facilitators (employer psychoeducation, job/career training supports) and accommodations (flexible hours/place, autism-friendly adjustments).	Modified TVI ranking: 49% working >10 h/week without support (rank 6); 43% with no vocational activities (rank 1); 8% intermediate ranks. Hours: Full-time n=114, Part-time n=45, Unemployed n=122.	Work readiness profile variegated: Work Habits strongest; Work Style (flexibility/adaptability) weakest. Logistic regression (AWSQ → vocational independence) accuracy 72.2% (vs 52.9% baseline); r²: Cox–Snell 0.25, Nagelkerke 0.34. Significant predictors: work style, level of independence, and routine daily activities; with covariates, work style remained significant; model accuracy was ~70.6–77% with covariates, a +6.5% gain when AWSQ was added. Group differences: Full-time > Part-time > Unemployed on all AWSQ scales (except Work Habits & Interpersonal Skills didn’t differ FT vs PT). Reported barriers: phenotype-related issues, job-search/fit challenges, and non-autism-friendly workplaces/burnout; facilitators mirror these (training, psychoeducation, and accommodations).
7	Zalewska et al., 2016 (JVR) [75]	Autism as a primary special education category (IDEA classification, consistent with DSM-IV/DSM-5 surveillance)	Varied; many in part-time, low-wage, disability-majority settings	Predictors examined: self-determination, social skills, job search, transportation independence	67% ever worked since HS; 45% currently employed; avg. 25.6 months at job; mean wage $7.70/hr; 23.7 hrs/week; fewer benefits; 34% “liked job very much.”	No significant association for overall self-determination, social skills, or job search. Psychological empowerment (subscale) predicted employment (R²=0.163, p<0.05). Transportation independence (driver’s license OR=5.2, p<0.01; independent travel OR=4.8, p<0.05) strongly predicted employment.
8	Martin et al., 2023 (Disability & Rehabilitation) [77]	A documented autism diagnosis required for supported employment services	Varied: food industry, manufacturing/distribution, media, IT, banking, healthcare, private and non-profit organizations	Supported employment with job coaches; job coaches facilitated hiring, onboarding, training, performance management, and communication between managers and autistic employees	Mixed: some maintained long-term jobs, others had contracts ended due to performance, organizational changes, or lack of support. Sustained employment is linked to high-quality manager-employee relationships and job coach mediation	The quality of the manager–employee relationship was the central factor for positive outcomes. Job coaches acted as relational mediators, fostering mutual understanding, clarifying expectations, and supporting adaptations. High-quality relationships led to pride, improved team climate, and employee growth. Poor or unsupported relationships led to termination. Highlights the Leader–Member Exchange (LMX) theory applied to autism employment.
9	Richardson et al., 2019 (Work) [78]	Autism Spectrum Disorder (clinical diagnosis; participants also identified as AAC users)	Varied employment sectors; jobs obtained through supported or customized employment	Focus on augmentative and alternative communication (AAC) in the workplace. Examined accommodations such as AAC device use, coworker support, job coaching, and flexibility in communication methods.	Adults with ASD + AAC were employed in community jobs; most maintained positions with ongoing support. Job satisfaction and inclusion varied.	Employment is possible but requires extra effort and flexibility from employers and families. Barriers: limited communication partner training, stigma, and low expectations. Facilitators: supportive supervisors, willingness to adapt tasks, strong advocacy by family/job coaches. The title theme “He’s worth the extra work,” reflects employer perceptions of employment as feasible but requiring tailored accommodations.
10	Katz et al., 2015 (Work) [79]	High-Functioning Autism Spectrum Disorder (clinical diagnosis; IQ >70 implied)	Open-market competitive jobs (varied sectors, matched after evaluation)	Structured work placement program: pre-assessment, tailored job matching, ongoing support by health professionals/team members, follow-up at 4 timepoints	100% job retention over 9 months; WPE (Work Performance Evaluation) stable-high and slightly improved; self-reported QoL increased significantly, esp. self-competency	Participants maintained jobs; both self and team ratings of performance improved slightly; QoL improvement was significant (greater sense of competence, p<0.05). The study highlights the importance of structured placement & support in sustaining employment.

Job Acquisition and Search Duration

The acquisition of a job and the duration of the search were the major issues. Studies of the nationally representative NLTS2 cohort [71] revealed that a mere 29% of autistic young adults were actively seeking employment at the time of interview, compared to 49-60% of their peers with other disabilities. Further, a mere 22% of autistic participants found employment independently, the lowest proportion among disability groups. The median job search was 14 months, more than double the two to 12 months commonly recorded in comparison samples. Successful acquisition was predicted by improved conversational ability and greater functional skills, both of which improved the probability of successful job-finding independently (OR=1.21, p < 0.01). Postsecondary education not only increased employment chances but also paradoxically reduced tenure, suggesting that a significant number of jobs did not align with qualifications. Further survey findings [70,73] supported these barriers, with burnout, job mismatch, and a lack of autism-sensitive recruitment strategies cited as the major obstacles. Young autistic adults, especially women and those with co-morbidities, were more likely to experience exclusion at the application stage. Overall, these results emphasize that gaining employment is still the most risky stage of the employment pathway, typically taking more time, multiple interventions, and specialist readiness strategies.

Retention and Job Stability

Retention and stability outcomes were more promising after autistic individuals achieved employment. Autistic employees in Wei et al. [71] kept jobs for an average duration of 23.5 months, longer than learning-disabled or speech-language-impaired peers. Katz et al. [79] also documented 100% retention in a structured work placement program with individually paced job matching and continued team support for nine months. These results indicate that autistic workers, put in nurturing settings, tend to show high levels of loyalty and long-term commitment. However, inequalities continued; the longitudinal autistic and non-autistic youths' study [72] revealed that a mere 3% of the autistic individuals worked full-time relative to 17% of their non-autistic counterparts. Discontinuities of contracts were frequently attributed to poor supervisor relationships or a lack of proper job support [77]. Stratton et al. [46] also demonstrated that co-occurring depression and disability days had higher rates of vocational impairment, resulting in less work continuity.

Job Satisfaction and Inclusion

Autistic workers tended to report satisfaction with their jobs, even when underemployed or working at a grade below their qualifications. Despite low salaries and poor benefits, nearly 90% of respondents in Wei et al. [71] indicated they enjoyed their work and received fair treatment. Harvey et al. [70] reported comparable trends: although 86% of the respondents worked, 37% were underemployed, and 51% underutilized in comparison to their qualifications. Fears of disclosure and unmet needs lowered perceived inclusion, yet where there were accommodations, satisfaction was greatly enhanced. Qualitative research better captured these dynamics. Richardson et al. [78] documented how AAC users were treated with dignity when supervisors modified communication practices, yet stigma and inadequate training decimated inclusion in some work environments. Martin et al. [77] similarly found that positive manager-employee relationships created pride, teamwork, and increased self-esteem among autistic workers. These results suggest that relational and environmental supports, rather than job type, heavily influence job satisfaction (Table 8).

Work Performance and Productivity

Work performance evidence was limited but uniform in indicating that autistic workers can function well when they have support. Katz et al. [79] reported stable-to-improving scores on the work performance evaluation (WPE) at four timepoints during a planned placement, together with significant improvements in self-reported quality of life. Similarly, Richardson et al. [78] observed that AAC accommodations reduced productivity, enabling the workers to play a significant role despite communication difficulties. The outcomes for employers were also observed in the RCT conducted by Scott et al. [69]: the use of the IEST™ toolkit enhanced employer self-efficacy (p=0.016, d=0.58), and in the case of frequent users, employer positive attitudes towards autistic employees should be translated into more effective support of employee productivity. However, Stratton et al. [46] cautioned that treatment of both health and workplace adjustments was essential since concurrent depression and disability days significantly impaired vocational functioning. In general, the data point in favor of the fact that productivity is not in itself limited by autism but rather is highly situational, support-based, and climate-sensitive.

Summary of Key Findings Across Studies

These ten studies have shown that there were several common trends in the effect of workplace accommodation on the employment of autistic individuals. First, it remained similar across the board that accommodations, be they in terms of technology, environment, supervisory, or psychosocial, were correlated with improved outcomes, namely job retention and job satisfaction (Table 8). Various designs of studies, including RCTs and longitudinal cohorts, demonstrated that accommodations could make the autistic workers much more likely to work longer, more satisfied with their jobs, and have a positive impact on productivity at work. These similarities contribute to the body of evidence by showing that the results of different kinds of support lead to one conclusion: that accommodations are the key to sustainable employment.

In contrast, significant differences and challenges were observed. One of the issues that happened over and over again was fear of disclosure, with autistic workers being hesitant to ask for accommodations because of stigma or fears of discrimination [70]. As a result, only a few participants received the workplace modifications they needed, despite several reporting unmet needs. Another difference was in supervisor awareness and quality of relationships. While Martin et al. [77] emphasized the strong role of positive manager-employee relationships in maintaining employment, other findings indicated the dangers of low-quality supervisory involvement, with contracts ceasing prematurely even when employees were capable. In addition, outcomes like job attainment and search length had varied findings: although autistic workers tended to have long waits to secure employment [71], they exhibited good loyalty and retention abilities upon being employed, particularly in formal programs [79]. Methodological variation between studies also influenced conclusions. Large-scale cohort examinations focused on systemic variables like transportation and education, while qualitative research elucidated the subtle interpersonal patterns of inclusion and stigma. These differences combined reinforce that the effect of accommodations is not consistent but context-specific, depending on individual, relational, and structural variables.

Discussions

Summary of Main Findings

This systematic review pooled evidence from ten empirical articles that appeared between 2015 and 2025 on workplace accommodations and employment outcomes for autistic adults. Across studies, a consistent picture emerged: accommodations of a technological, environmental, supervisory, or psychosocial nature were broadly linked with positive outcomes, particularly in job retention, satisfaction, and productivity (Tables 3, 4). While the methodological quality was overall high to moderate, the majority of the studies used self-reported information, cross-sectional study designs, or secondary analyses, which precluded inferences of causality.

Psychosocial and skills-based supports showed the most robust evidence. For instance, Katz et al. [79] exhibited 100% job retention in a formal placement program, whereas Wei et al. [71] established that conversational and functional skills were predictors of longer job tenure and greater independence in job acquisition. These results are supported by large-scale intervention studies that indicate that individualized and supported employment schemes enhance retention as well as pay [80,81]. Environmental and organizational adjustments, like flexible timetables, sensory accommodations, and transport independence, were also repeatedly associated with greater utilization and satisfaction [70,75]. This result is consistent with scoping reviews, which emphasize that environmental fit plays a critical role in autistic workers' sense of belonging [82,83].

Evidence regarding supervisory and relational support similarly supports the significance of social context. Both Scott et al. [69] and Martin et al. [77] demonstrated that job coaches and supportive managers improved job stability, reflective of wider literature highlighting the importance of relational quality in maintaining jobs [84,85]. Conversely, the category of assistive technology was the least diverse, with only Richardson et al. [78] reporting on it, although other trials, like Gentry et al. [86], reported that digital support was associated with reduced reliance on one-to-one support. In spite of such consistencies, principal challenges were highlighted. Resistance to disclosure restricted access to accommodations [70], lack of awareness among supervisors compromised otherwise talented staff [77], and comorbid mental health challenges compromised vocational functioning [74]. Such findings reflect larger systematic reviews reporting inconsistent implementation, small sample sizes, and limited diversity in autism studies of employment [87,88].

Interpretation of Findings

The synthesis of data from the included research reveals a similar trend: workplace accommodations correlate with improved job prospects for autistic individuals. At the same time, the dynamics of this association are multifaceted and influenced by many underlying factors. Supports were not consistently advantageous across all situations; rather, their correlations with outcomes fluctuated based on the interplay of individual competencies, the quality of interpersonal interactions, and the overarching organizational environment.

One mechanism is likely the relational aspect of work. Martin et al. [77] illustrated that supervisor-employee relationship quality was key to long-term employment, where job coaches served as relational mediators. Likewise, Scott et al. [69] revealed that the IEST™ employer training toolkit enhanced supervisors' self-efficacy and attitudes towards autistic employees, implying that employer confidence and awareness are preconditions for successful accommodation. This has support from Nicholas et al. [84], who demonstrated that employment-support services are most beneficial when they focus not just on the autistic employee's abilities but on the practice and attitude within the workplace setting. The evidence together indicates that accommodations are effective less by "curing" the autistic person and more by redesigning the social environment to include them.

Environmental and organizational accommodations, including sensory modification, flexible scheduling, and transportation autonomy, came forward as another strategy through which accommodations ensure success. Harvey et al. [70] and Zalewska et al. [75] both reported that access to such modifications greatly enhanced proper job placement and employment levels. These modifications may lessen the effects of environmental stresses that are often associated with sensory overload or challenges stemming from rigid scheduling. Wider work supports this, with Pfeiffer et al. [83,89] demonstrating that person-environment fit was a strong predictor of job satisfaction and performance for autistic staff. In doing so, environmental adaptations do more than render work manageable; they open up the potential for staff to work at their real level.

Skills-based and psychosocial treatments were observed to correlate with enhanced readiness and self-efficacy. Katz et al. [79] discovered that structured placements with tailored job matching correlated with elevated retention rates and enhancements in self-reported quality of life, but definitive causal inferences cannot be established from the research design. Wei et al. [71] demonstrated that functional and conversational skills could predict both tenure at work and the acquisition of sole employment. These results demonstrate that the development of adaptive and communication skills prepares autistic workers to engage in hiring and retention processes more efficiently. Concurrent evidence from intervention studies [81,90] also indicates that specialized vocational readiness training, e.g., virtual interview training or supported internships, can have a strong effect on hiring and wage outcomes. The mechanism in this case is in building confidence and autonomy and, in the process, weaning from external supports [86].

Meanwhile, several studies have highlighted ways in which barriers defeat these mechanisms. Concerns about disclosure, as recorded by Harvey et al. [70], stop employees from asking for required modifications, thus obliterating the possible advantages of accessible supports. Stratton et al. [46] similarly found that depression and disability days predicted poorer vocational functioning, suggesting that comorbid mental health conditions may undermine otherwise effective accommodations. These results reflect more general literature that identifies poor person-environment fit with mental health challenges [91,92]. Most relevantly, they portray the achievement of accommodations as dependent not just on structural availability but on psychological safety and health as well.

Together, these processes imply a rethinking of how accommodations are to be understood. Instead of being viewed as peripheral modifications, they are dynamic interventions that intervene between personal attributes and systemic obstacles. Accommodations that reform the social context (supervisory training), alter the physical and organizational environment (sensory and scheduling adaptability), and enhance individual capabilities (readiness and communication assistance) work synergistically to create lasting employment outcomes. The breakdown of accommodations, on the other hand, usually results not from an inherent flaw but from inadequate disclosure, ineffective implementation, or insufficient employer preparedness.

Comparison With Previous Literature

The results of this review correspond to, but also complement, the wider literature on employment and autism. The ten included studies uniformly correlated accommodations with positive outcomes in job obtaining, job maintenance, and job satisfaction. This aligns with previous systematic reviews and scoping syntheses that have emphasized the helpful contribution of individualized support for autistic individuals at work [82,88]. But the current review contributes to knowledge by synthesizing and critically combining experimental and observational data, demonstrating not just whether accommodations are effective but also how they operate within various organizational settings.

One obvious point of overlap with existing research is the place of supported employment models. Early randomized controlled trials by Wehman et al. [80,81] proved that structured internship models like Project SEARCH, combined with on-the-job coaching, greatly enhanced employment outcomes and wages. The present review corroborated these findings, with Katz et al. [79] and Nicholas et al. [84] further showing that employer involvement and vocational matching maximize long-term retention. This adds to previous evidence collated by Harmuth et al. [87], who drew the conclusion that supported employment is still the most strongly evidenced intervention for autistic people, especially if employer training is included. Our synthesis thus reaffirms the general view that long-term, systematic support models are more effective than ad hoc or transient approaches.

The results also cross over with assistive technology studies. Gentry et al. [86] offered experimental proof that handheld digital technology might decrease the use of personal job supports to facilitate higher autonomy. This study concurred with Fletcher-Watson [93], whose systematic review of computer-assisted learning likewise stressed that technology can scaffold autonomy and skill generalization. Nonetheless, whereas previous literature tended to examine cognitive and instructional consequences, the research contained within this review illustrates a clear translation of assistive technology into workplace productivity and viability. Thus, the current results extend the technological account into applied vocational arenas, closing a gap between educational and work-based streams of inquiry.

Another area of convergence in past scholarship was the significance of environmental and organizational adaptations. Pfeiffer et al. [83,89] had shown that person-environment fit was a strong predictor of satisfaction and performance among autistic workers, a note sounded in several of the studies presented herein. The ubiquity of this observation across environments implies that accommodations within the environment, like sensory adjustments or adjustable scheduling, are not secondary but essential for successful work. This observation is in line with wider disability studies, where workplace adaptability and design have been known for a long time as inclusion determinants [94]. However, the autism-specific research fills the gap by demonstrating how such adaptations actually mediate sensory sensitivities and executive functioning difficulties directly, thus applying universal principles of workplace accessibility to neurodiverse groups.

Where the current review breaks with previous literature is the prioritization of relational quality and supervisory support. Although previous reviews [82,85] referred to supervisory attitudes as a hindrance, they often regarded these as secondary factors. In contrast, our synthesis, specifically based on Martin et al. [77] and Swarbrick et al. [95], prioritizes supervisory relationships as accommodation mechanisms of first importance. Supervisory support was not just about following policy but about creating trust, communication, and inclusion. This study broadens the literature on neurodiversity [88], which positions workplace culture as a key driver of success, beyond the offering of individualized support. Therefore, current findings suggest a shift in emphasis: we should consider relational and cultural accommodations as crucial as technological or environmental ones.

The review also identifies areas of variation and inconsistency in relation to previous evidence. For example, although Smith et al. [90] observed significant interview skill improvement after virtual reality training, some studies revealed that fear of disclosure [70] and mental illness [74] would restrict the transfer of skills to actual outcomes. This issue is echoed by a general tension in the literature between skill use and skill acquisition, with the implication that successful accommodations need to straddle both areas. Likewise, though follow-ups at longer intervals within Moss et al. [91] identified ongoing mental health challenges within autistic adults, numerous intervention studies included here did not incorporate adequate mental health support. This gap reflects a disparity in research priorities, where vocational interventions seldom cater to co-occurring conditions even though their effects on employability are well established.

Thirdly, this review's findings also supplement general disability employment research. Lindsay et al. [94] showed that employing individuals with disabilities translates into organizational gains, such as enhanced workplace culture and innovation. The present review supports this by demonstrating that well-designed accommodations not only improve the performance of autistic staff but also construct inclusive practices of benefit to entire organizations. Thus, the evidence speaks in favor of the "business case" for neurodiversity set forth in recent management discourse and links social inclusion with concrete organizational benefits.

Discrepancies and Limitations in the Evidence

While the overall evidence base shows that workplace accommodations are positive for autistic workers, some discrepancies and limitations moderate the robustness of conclusions. These are issues both reflecting methodological limitations across included studies as well as larger structural gaps within the field. One primary limitation relates to variability in measurement strategies. Across the ten studies, outcomes varied from rates of job acquisition and tenure duration [80,81] to subjective satisfaction ratings [83,89] and qualitative reports of relational supports [95]. Such heterogeneity makes direct comparison difficult and precludes aggregation through meta-analysis. For instance, while Nicholas et al. [84] offered detailed service-level ratings, Smith et al. [90] considered interview skills exclusively. Without agreed-upon outcome measures, it is still challenging to ascertain which types of accommodation have the most potent or long-lasting effects. This feature resonates with worries voiced by Harmuth et al. [87] that autism work literature is absent of shared metrics, eroding cross-study synthesis.

Another limitation is that samples tend to be small and selective. Several trials had fewer than 50 participants, with Wittevrongel et al. [96] and Scott et al. [97] basing their evidence on very local cohorts. Even in larger trials, like those by Wehman and colleagues [80,81], participants were frequently recruited from school-to-work transition programs, limiting generalizability to older adults or individuals outside structured services. In addition, many samples were biased towards White individuals, city dwellers, and more capable populations, with under-representation of ethnic minorities, rural communities, or those with co-occurring intellectual disabilities. This population homogeneity mirrors longstanding criticism in autism studies more generally [85] and limits the external validity of findings to the broader autistic population.

A further consistency concern is the presence of reliance on self-report or proxy-report data. Research assessing environmental fit [83] or relational quality [77] often measured outcomes through questionnaires that are subject to social desirability bias, recall error, or restricted self-awareness. Although self-report accounts for subjective experience, a key component of workplace inclusion, it is not always matched with objective measures like earnings, job tenure, or productivity. The gap between perceived and observed outcomes has also been highlighted in associated literature on neurodiversity at work [88], implying the necessity of multi-method solutions integrating subjective and objective measures.

There is also a disparity in incorporating mental health considerations. Although some research, for example, Stratton et al. [46], directly recognized that anxiety, depression, or post-traumatic stress disorder (PTSD) may negate vocational gains, most interventions isolated work from other areas. However, longitudinal follow-up data [91] indicate that co-occurring mental health problems greatly influence employability. The omission of combined mental health assessments is therefore both a methodological and conceptual shortcoming in intervention design.

The time horizon of research also limits confidence in the findings. The majority of interventions were measured at brief follow-up periods, three to twelve months following implementation. Few (e.g., Taylor et al. [98]; Wehman et al. [81]) followed longer-term pathways. This raises questions of whether the improvement seen is maintained for years or decades down the line, especially amid work setting changes, economic recessions, or shifts in employer leadership. More general disability employment studies [94] have recognized longitudinal gaps as a priority topic, and they continue to play a pivotal role in autism-specific studies.

Finally, inconsistencies in the level of employer engagement were evident across the studies. While Wehman et al. [81] and Kaufmann & Chang et al. [99] involved employers as active stakeholders in the delivery of interventions, others depended more on job coaches or family supports external to the employer [95]. This difference is a reflection of uncertainty regarding the best mix between internal organizational capabilities and external services. Absent more precise comparative information, it is difficult to say whether employer-sponsored or externally funded models yield more sustainable results.

Considered as a whole, these differences indicate that the extant evidence base, though promising, is piecemeal. Small, selective samples, varied outcome measures, self-reported dependence, brief follow-ups, and uneven employer participation all together restrict the generalizability and interpretability of the results. These methodological limitations place emphasis on existing evidence as indicative and not definitive, and they provide the context for establishing practical and policy implications as well as research priorities for the future.

Implications for practice and policy

The conclusions drawn from this analysis have significant ramifications for the planning, execution, and maintenance of accommodations for autistic personnel by employers, clinicians, and politicians. The overall message is clear: adjustments always lead to better results, but the details show that a multi-level, systems-oriented approach is needed for effective practice.

Employer Practice and Workplace Culture

The results underscore that employers should not view accommodations as optional extras but rather as essential components of an accessible workplace design. Environmental accommodations like flexible schedules, quiet rooms, and sensory adjustments were consistently associated with heightened satisfaction and performance [83,89]. Employers should thus integrate such practices into organizational policy, as opposed to depending on personal disclosures to initiate accommodations. This accords with the neurodiversity model, which positions workplaces as having an obligation to create settings in which diverse workers can function [88]. Just as critical are supervisory and relational supports. Martin et al. [77] and Swarbrick et al. [95] demonstrated that family advocates and managers play key roles in maintaining employment. Thus, employers should invest in supervisor training to enhance their understanding of autistic communication styles, strengths, and support requirements. Policies need to shift from compliance-based disability awareness modules toward relational capacity-building, providing supervisors with competencies to establish trust, provide constructive criticism, and manage disclosure sensitively.

Clinical and Vocational Services

Vocational support providers and clinicians also have key roles to play. Evidence from supported employment [80,81,84] suggests that highly structured, skills-focused interventions can have a profound impact. Clinical teams should incorporate job coaching, social skills training, and technology-enabled interventions like virtual reality interview practice [90] into rehabilitation services. Furthermore, given the extensive documentation of co-occurring mental illnesses [74,91], it is imperative to provide vocational interventions in multidisciplinary settings that incorporate psychological interventions. Even well-crafted accommodations are unlikely to result in long-term improvements unless they address anxiety, depression, and trauma.

Policy Implications

From a policy perspective, this review highlights the necessity for legislative and funding environments that promote and normalize accommodations. Initiatives like Project SEARCH demonstrate how employer-driven collaborations, informed by policy funding, can enhance autistic youth's long-term results [81]. Policymakers would thus increase funding for supported employment models, offer tax benefits to inclusive hiring, and tighten legal barriers to workplace discrimination. Accommodation should also be established as a legal right in addition to economic and social investment. Research, including Lindsay et al. [94], has determined that the employment of people with disabilities is cost-effective and yields organizational and social returns, including high productivity and innovation. These facts, included in the policy wording, will shift the emphasis from compliance to opportunity.

System-Level Recommendations

Finally, the evidence suggests that meaningful accommodation requires coordination among systems. In between clinical services and employers, supported employment cannot be separated. Instead, they should be enabled by government-service provider-advocacy-business liaisons. Kaufmann & Chang et al. [99] have some hope for their end-to-end workplace program that connects recruitment, training, and continued support. Such scaling models require cross-sectoral purchasing and long-term investing.

Strengths and limitations

The systematic review contributes to the literature on autism and its work by providing a synthesis of a heterogeneous body of research performed using a range of different experimental, cohort, and observational designs across countries. One of the strongest points is the fact that the inclusion criteria are broad. The review adopts a broad perspective on the functioning of accommodations in various situations by including randomized controlled trials (e.g., Wehman et al. [80,81]; Gentry et al. [86]) and real-world cohort and survey studies (e.g., Nicholas et al. [84]; Pfeiffer et al. [83,89]. The ecological scope of such is valid, indicating that accommodations are beneficial not only in the highly controlled interventions but also in the naturalistic work environment.

A second strength is attention to more than one accommodation domain: assistive technology, organizational/environmental supports, supervisory/relational interventions, and skills/psychosocial programs. Earlier reviews typically highlighted just one category, e.g., supported employment [87] or environmental fit [82]. Discussing several categories simultaneously, the current review calls out how accommodations work synergistically. Technology, for instance, can maximize independence, but supportive relationships and organizational environments are just as important in maintaining inclusion.

The review is also enhanced by a systematic quality assessment with validated instruments (CASP, JBI, RoB). The majority of included studies were rated as being of moderate-to-high quality and thus lend strength to the overall findings. Notably, the synthesis goes beyond mere outcome reporting to tease out underlying mechanisms like relational trust and organizational culture that drive successful and unsuccessful accommodations.

Strengths aside, there are several limitations to be addressed. First, the heterogeneity of studies included design, outcome measures, and population precluded the possibility of a meta-analysis. Therefore, the results must be treated as a narrative synthesis and not quantitative pooled estimates. Second, the geographic distribution of studies was not balanced. Most of the evidence came from North America and Europe, with very little from low- and middle-income nations. This limits the applicability of results to international settings where resources and cultural perceptions of disability are distinct.

A further limitation relates to the reporting quality in primary studies. Most were based substantially on self-report measures, with few using objective indicators such as wages, job tenure, or performance rating. Moreover, most studies were of brief duration, raising questions about the long-term maintenance of effects. In addition, demographic representation was limited: few studies reported participants' ethnicity, socioeconomic status, or co-occurring intellectual disabilities at all. Therefore, the evidence base is probably based on a relatively affluent subgroup of autistic adults, which gives rise to equity and inclusion concerns.

Lastly, a weakness of this review itself is the breadth of available evidence. Ten studies were included under the inclusion criteria, and while they were useful, the number is small enough to limit the ability to generalize. Moreover, while attempts were made to reduce bias through systematic approaches, the lack of autistic people's participatory involvement in the review process may have limited the construction and interpretation of findings.

Directions for future research

This review focuses on the advancements achieved in autism and employment research while also underscoring the considerable deficiencies that persist. Future research should concentrate on the standardization of outcome metrics, longitudinal and large-scale methodologies, the inclusion of underrepresented populations, the integration of mental health and co-occurring conditions, the mechanisms of accommodation effectiveness, employer engagement, organizational transformation, and participatory frameworks. Standardized outcome indicators would improve comparability and facilitate the collection of substantive information. Longitudinal studies are crucial to analyzing the efficacy of accommodations over various career paths, during transitional phases, and in response to fluctuating economic situations. There is a notable deficiency of research in low- and middle-income nations, despite indications that cultural perceptions of disability and workplace infrastructure exhibit significant variability.

Integrating mental health and co-occurring problems is essential, since several employment-focused therapies have neglected these aspects. Subsequent studies ought to implement integrated methodologies that assess both occupational and mental health results concurrently. A comparative study is necessary to ascertain whether outcomes vary when accommodations are integrated into organizational culture as opposed to being provided by external entities. Participatory and co-produced research must prioritize autistic voices, guaranteeing that research meets genuine needs and mitigates the likelihood of creating therapies that conflict with employee interests.

## Conclusions

This review rigorously synthesized evidence on workplace accommodations for autistic adults. Three major conclusions arose: accommodations within technological, environmental, supervisory, and psychosocial areas tended to be associated with improved employment outcomes; relational and organizational support were particularly powerful in supporting maintenance of inclusion; and ongoing barriers of disclosure, stigma, and awareness on the part of supervisors constrained impact. Methodological gaps, small sample sizes, and the under-representation of diverse groups, however, limit generalizability. Future studies must focus on longitudinal trials, participatory design, and inclusive sampling to advance practice and provide equitable, sustainable work opportunities for the autistic population.
